# Gonorrhœa

**Published:** 1879-10-20

**Authors:** H. M. Lawson

**Affiliations:** Georgia


### *GONORRHŒA.*

BY DR. H. M. LAWSON, OF GEORGIA.

Several having inquired how I use the bandage spoken of in my little article on the treatment of gonorrhœa in the August number of the Journal, please allow me to reply through the Record, as others may desire the same information.

The bandage is used in this manner:

Take a bandage three or four feet long, one inch wide; split one end six inches; wrap the penis from tip to pubis; cross the tails behind the scrotum, and tie above the penis in front.

Before the bandage is appled (I use it only at night in these cases), my instructions are: Urinate, inject, apply the bandage at bed time.

2d. What proportion of guaiac and rosin do you use and in what dose?

℞ Red rosin........................................ ℥ij.
Gum guaiac........................................ ℥ss.

Reduce to coarse powder, add one quart of gin and allow to stand twelve or twenty-four hours. Dose, a tablespoonful three times a day.

Use only the hard, red rosin found around old scars, other kinds are of little value.

3d. What injection is best?

While in the army I had to use chloride of zinc, having nothing else; finding it sufficient for most cases, I still use it, except where other injections are indicated. As a general thing, any of the usual injections will answer, if of the proper strength.

I use the zinc thus:

℞ Chloride zinc.................................... gr. j to iij.
Aqua fontis...... ................................ ℥j.

In patients suffering for the first time, one gr. to the ounce is sufficient, but if the disease has become chronic, two grains to the ounce is used. In patients who have had the disease once, or oftener, before, I use two grains to the ounce, if the case is recent; three grains, if chronic.

The injections must be used six or seven times a day for two days (see page 290 of Record), then put the patient upon the rosin and guaiac, and nothing else, and I have yet to see the first case that resists the treatment.

I make my patient use the syringe with water before he leaves my office, until he can use it properly; this is important. I also apply the bandage for him the first time myself.

